# “Beyond laughter”: a systematic review to understand how interventions utilise comedy for individuals experiencing mental health problems

**DOI:** 10.3389/fpsyg.2023.1161703

**Published:** 2023-08-07

**Authors:** Eshika Kafle, Cat Papastavrou Brooks, Dave Chawner, Una Foye, Dieter Declercq, Helen Brooks

**Affiliations:** ^1^School of Arts, University of Kent, Canterbury, United Kingdom; ^2^Sussex Partnership Innovation and Research in Eating Disorders (SPIRED) Clinic, Sussex Partnership Foundation Trust, Sussex, United Kingdom; ^3^Bristol Medical School, University of Bristol, Bristol, United Kingdom; ^4^Department of Mental Health Nursing, King's College London, London, United Kingdom; ^5^Mental Health Research Group, Division of Nursing, Midwifery and Social Work, School of Health Sciences, Faculty of Biology, Medicine and Health, University of Manchester, Manchester, United Kingdom

**Keywords:** mental health, mental illness, recovery, CHIME, comedy, humour, comedy intervention, humour intervention

## Abstract

**Introduction:**

There is evidence for the impact of comedy and humour for mental health and wellbeing. Existing systematic reviews have concluded laughter has a positive impact on wellbeing, however other potential benefits of comedy interventions have remained under explored. The aim of the current study was to synthesise current evidence for comedy/humour interventions and evaluate mechanisms through which comedy interventions may impact upon the recovery of those experiencing psychological distress, using the Connectedness, Hope, Identity, Meaning and Purpose and Empowerment (CHIME) framework.

**Methods:**

Five electronic databases were searched for studies exploring the impact of interventions using comedy on wellbeing and mental health recovery, from earliest record until January 2023. Grey literature was obtained via contacting experts in comedy interventions for mental health and supplemented by an internet search for comedy interventions. To be eligible for inclusion, studies had to include primary data, published in English or German, and explore a population of adults, with self-reported distress or a self-reported/diagnosed mental health condition. Studies included only explored interventions which utilised comedy as the main intervention and aimed to induce ‘simulated’ laughter, in response to a stimulus. 17 studies were included in the review.

**Results:**

Studies were found to have positive impact on mental health symptoms and several mechanisms of the CHIME framework for recovery, including connectedness, hope, identity and empowerment. Potential theorised mechanisms for change included confidence in new skills, promotion of social skills, opportunities for social interaction, laughter, vulnerability, and cognitive flexibility. The current review found that comedy/humour interventions are beneficial for mental health recovery and wellbeing and found preliminary evidence for a range of mechanisms through which comedy may have positive impact.

**Discussion:**

Further research should focus on qualitative exploration of the mechanisms by which comedy interventions may have impact on wellbeing and mental health recovery for specific populations and within different settings. It is concluded that there is a need for transdisciplinary collaboration in research on comedy interventions, which brings together the expertise of comedians delivering/developing interventions, those with lived experience of mental health issues and researchers from both health sciences and humanities disciplines.

## 1. Introduction

Mental health difficulties are highly prevalent, with estimated occurrence rates of between 18 and 36% globally (Kessler et al., 2007). The experience and outcomes of mental distress are highly individualised but the impacts are considered long-lasting and wide ranging (Ferrari et al., 2014) Recent evidence suggests that statutory mental health services are failing to provide care that adequately addresses individual needs and preferences (Bee et al., 2015). Reasons for this include a focus on dyadic interactions with health professionals to the detriment of considering the role of wider networks of support including engagement with valued activities (Slade, 2010). This has led to recent calls for greater consideration to be given to alternative forms of therapeutic provision for people experiencing mental health difficulties (Ostermann et al., 2019; Turgon et al., 2019).

The idea that “laughter is the best medicine” is part of folk psychology – and has crystallized into a well-known proverb. Over the years, this idea has been subject to a considerable amount of academic scrutiny (Fry, 1994; Lefcourt, 2001; Martin and Kuiper, 2016), including useful nuances and distinctions between adaptive and maladaptive humour uses (Martin et al., 2003; Kuiper et al., 2004). However, laughter is meaningfully distinguished from humour. Laughter is a physical response that is not exclusively elicited through humour and non-human animals laugh for reasons that are very different from the perception of humour (Mathevon et al., 2010) – although such non-human laughter, from an evolutionary perspective, may be very distinct from human laughter, which derives from primate play (Gervais and Wilson, 2005). In this respect, health interventions like Laughter Yoga, which involves prolonged voluntary laughter, are different from interventions like stand-up comedy workshops, where laughter is elicited in response to humour (van der Wal and Kok, 2019).

Laughter is also not a necessary response to the perception of humour; one can simply smile at a joke. In other words, laughter is neither a necessary nor a sufficient condition for humour. Instead, humour is more usefully framed as the object of an emotional state, i.e., comic amusement (da Silva, 2018) or mirth (Martin, 2007). Humour, as the phenomenon which elicits amusement, can also be distinguished from sense of humour, i.e., a disposition for humour use (Martin et al., 2003) or character strength (Müller and Ruch, 2011).

Humour has been framed as a resource to increase resilience and promote wellbeing by allowing cognitive reappraisal of negative life events (Kuiper, 2012) and to help re-frame challenging situations like a cancer diagnosis as less threatening (Demjén, 2016). Development of humour may therefore be beneficial to mental health recovery, particularly depression, which has been thought to involve a negative information processing bias (Beck and Clark, 1988; Declercq, 2021). However, despite preliminary evidence for the benefits of humour on health, the field remains largely underexplored (Fischer et al., 2021).

Existing systematic reviews have concluded that there is evidence for positive benefits of laughter inducing therapies on wellbeing and mental health (Bressington et al., 2018; Gonot-Schoupinsky and Garip, 2018; van der Wal and Kok, 2019). Comedy may impact wellbeing through a wide range of mechanisms, aside from laughter, including cognitive reframing (Kuiper, 2012) and distancing through the positive emotional qualities of amusement (Folkman and Moskowitz, 2004) and broadening and building cognitive resources (Fredrickson, 2004; Gervais and Wilson, 2005). However, few systematic reviews have evaluated the potential benefits of comedy interventions which aim to improve wellbeing via humour/amusement, rather than simulated laughter. Linge-Dahl et al. (2018) found that humour had a positive impact on patients, families and carers in palliative care. However only two studies, included in the review, evaluated the impact of humour interventions. Similarly, Gonot-Schoupinsky and Garip (2018) found limited evidence, from only one study, for the impact of an intervention which utilised humour-induced laughter, in their review on the impact of laughter and humour on wellbeing of older adults.

Despite preliminary evidence for the impact of humour and comedy interventions for wellbeing, the impact of these interventions on those living with mental health issues remains underexplored. It is possible that the focus on laughter as the mechanism for comedy’s impact on wellbeing, prevents comprehensive understanding of the ways in which humour and comedy can aid the wellbeing of those in psychological distress.

Furthermore, there has been a recent call towards focusing on the importance of ‘personal recovery’, as opposed to ‘clinical recovery’, defined as living a life without symptoms of mental illness (Andreasen et al., 2005) in treatment for mental health issues (Liberman and Kopelowicz, 2005; Roe et al., 2011). Personal recovery is defined by living a satisfying and meaningful life, whilst living with or without symptoms of the mental health disorder (Anthony, 1993). Personal recovery has been conceptualized using the Connectedness, Hope, Identity, Meaning and Purpose and Empowerment (CHIME) framework (Leamy et al., 2011; Van Weeghel et al., 2019). The five components of CHIME indicate different aspects of personal recovery. These are outlined in Table 1. The CHIME framework has been validated for use cross-culturally (Brijnath, 2015; Apostolopoulou et al., 2020; Vogel et al., 2020). Use of the CHIME framework can allow for ‘personal recovery facilitators’, which refers non-traditional therapeutic interventions that aim to support personal recovery (Isaacs et al., 2022) such as comedy, to be evaluated as recovery focused approaches. CHIME has been used to explore the impact of art and music therapies on mental health recovery (Damsgaard and Jensen, 2021). However, no research has examined the impact of comedy interventions on aspects of CHIME. The present review aims to explore the mechanisms through which comedy interventions can impact upon the recovery and wellbeing of those experiencing psychological distress, using the CHIME framework.

**Table 1:** CHIME Components.

Component	Sub-components
Connectedness	• Peer support and peer groups • Relationships • Support from others • Being part of the community
Hope and optimism	• Belief in possibility of recovery • Motivation to change • Hope-inspiring relationships • Positive thinking and valuing success • Having dreams and aspirations
Identity	• Dimensions of identity • Rebuilding or redefining a positive sense of identity • Overcoming stigma
Meaning in life	• Meaning of mental illness experiences • Spirituality • Quality of life • Meaningful life and social roles • Meaningful life and social goals • Rebuilding life
Empowerment	• Personal responsibility • Control over life • Focusing upon strengths

Derived from Leamy et al. (2011).

The aim of the current review is to evaluate the evidence on comedy interventions for mental health recovery including:

1. The impact of comedy interventions for those experiencing mental health issues and psychological distress.
2. The mechanisms of impact for comedy interventions for those experiencing mental health issues.

## 2. Methods

Five electronic databases were searched initially in January 2022, searches were updated in January 2023. Methods and results were reported according to PRISMA (Preferred Reporting Items for Systematic Reviews and Meta-Analyses) guidelines (Page et al., 2021). Unpublished literature was also sought out and included in this review.

### 2.1. Eligibility criteria

Studies reporting primary data, published in English or German, which examined the use of comedy interventions to improve mental health were eligible for inclusion. No restrictions were placed on publication date. Inclusion/exclusion criteria can be found in Table 2. Any study design, including qualitative, quantitative and mixed methods were included, including unpublished studies.

**Table 2:** Inclusion and exclusion criteria.

Inclusion criteria	Exclusion criteria
Quantitative, qualitative, mixed methods, published and unpublished studies	Duplicates
Studies which include primary data, published in English or German.	Case studies, studies not published in English, conference abstracts and manuscripts only available in abstract form.
Adults aged over 18, with a diagnosed or self-diagnosed mental health disorder. To be included in the review, studies must include a minimum of 75% of people with a diagnosed or self-reported mental health difficulty.	Studies with populations aged under 18.
Studies which investigate those with self-reported or diagnosed substance abuse, mood disorder, eating disorder, anxiety disorder, psychotic disorder.	Studies which include populations with a neurodevelopmental disorder, such as ADHD, Autism, dementia.
Interventions which use comedy, amusement and humour to benefit those experiencing mental health issues.	Interventions of which the only aim/mechanism of action is to induce ‘simulated laughter’, which is laughter not in response to a stimulus. For example, laughter yoga.
Studies which utilise humour/comedy as the main intervention.	Studies which use humour as part of delivery of another intervention, e.g., therapist use of humour in therapy.
	Studies which use humour/comedy as one component of intervention, e.g., use of humour in drama therapy, play therapy
Studies must include a mental-health related outcome (including known contributors to mental health)	Studies which do not include a mental health-related outcome (including known contributors to mental health, e.g., social connectedness)

Papers were included if a minimum of 75% of their sample comprised of those aged over 18, experiencing a diagnosed or self-reported mental health problem, including psychological distress. Those experiencing neurodevelopmental conditions such as Attention Deficit Hyperactivity Disorder (ADHD), dementia and Autism were not be included in the review, unless co-occurring with another diagnosed mental health condition or psychological distress.

Studies were eligible for inclusion if they reported on an intervention in which comedy/humour was the core component. Studies were excluded if comedy/humour was used as part of delivery of a primary intervention or if they utilised comedy/humour only as one component of an intervention, e.g., use of humour in drama therapy, play therapy.

Only studies which aim to induce amusement or ‘spontaneous’ laughter (Mora-Ripoll, 2011; Yim, 2016) were included in the study. Spontaneous laughter occurs in response to a stimulus. Studies included reported mental health-related outcomes. Reviews and articles only in abstract form were excluded from the review.

#### 2.1.1. Search strategy

Electronic database searches were conducted of Medline, AMED, Psychinfo & Embase, Web of Science and Google Scholar in January 2022 and updated in January 2023. Grey literature was searched for through contacting experts who deliver comedy interventions for mental health. A database of experts in comedy interventions for mental health was compiled by authors and supplemented by an internet search for comedy interventions Experts were contacted by the authors to obtain reports evaluating any comedy interventions they had conducted. Reference sections of eligible papers were also manually searched and relevant papers screened for eligibility.

**Figure 1:**
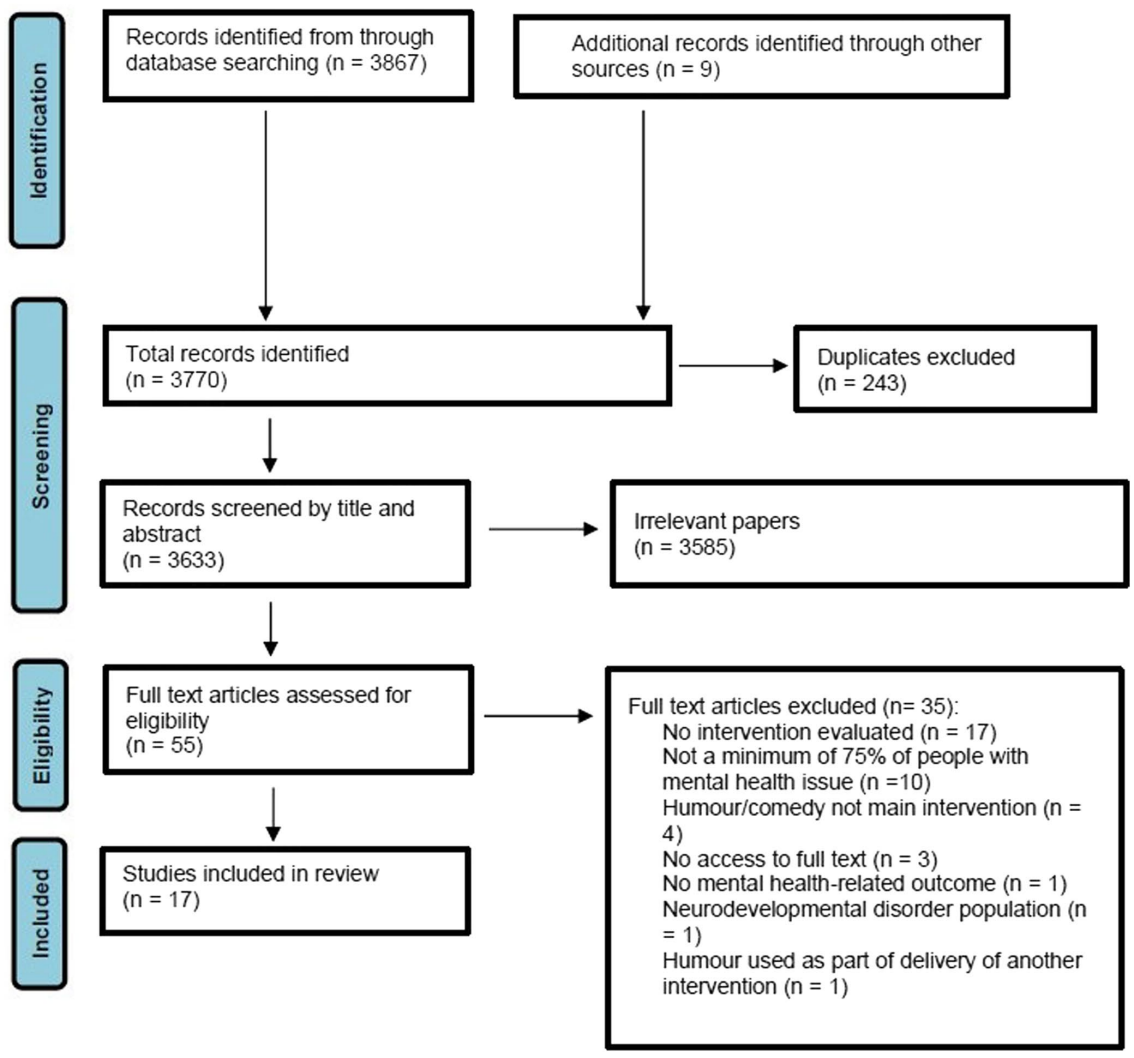
PRISMA flow diagram.

Search terms related to comedy interventions were combined with search terms related to mental health disorders. Agreed search terms were combined using the Boolean operator ‘OR’ and across components using ‘AND’. Search terms were reviewed and amended by a research librarian, from Sussex Partnership Foundation Trust. A full search strategy is included in Supplementary Data Sheet 1. PRISMA flow diagram can be found in Figure 1.

### 2.2. Review strategy

Search results were uploaded to Covidence.^1^ Duplicates were removed automatically via Covidence. Firstly, each study’s abstract and title was reviewed for inclusion, against pre-set criteria, independently by two researchers. Full texts of eligible studies were then also screened independently by two reviewers. Any conflict was resolved via discussion amongst reviewers to reach a consensus. Any conflicts not resolvable by consensus were referred to a third party.

### 2.3. Data extraction

The majority of data were extracted from included studies by one researcher out of a team of seven. 30% of studies were reviewed independently by a second researcher. Data were extracted using a form developed collaboratively within the research team. Data were extracted on study characteristics, intervention characteristics, population details, study outcomes (such as positive/negative impacts of intervention, mechanisms of action, acceptability of intervention and experiences of intervention). The lead researcher went through all researchers’ extraction tables. If any minor discrepancies occurred, this was discussed in regular meetings between all authors. If there were major discrepancies, it was decided that the extraction framework would be reviewed. However, this was not necessary. The CHIME (connectedness, hope and optimism, identity, meaning, empowerment) framework (Leamy et al., 2011) for mental health recovery was utilised to help identify key mental health-related outcomes of comedy interventions.

### 2.4. Quality assessment

The MMAT quality appraisal tool (Hong et al., 2018) was used to assess study quality. Quality assessment was carried out by one researcher, with 30% of included assessments checked independently by a second researcher. Any disagreements were resolved through discussion or via referral to a third party.

### 2.5. Data synthesis

A narrative synthesis approach to analysing, integrating and synthesising findings, using Popay’s et al. (2006) guidance on the use of narrative synthesis in systematic reviews of effectiveness studies, was used. This involved tabulation of study characteristics and extracted data (supported by Microsoft Excel) and a narrative synthesis of extracted data which was undertaken over four stages: (i) Developing a theory of how comedy interventions work (ii) Developing a preliminary synthesis, undertaken by organising extracted tabulated data into clusters (e.g., intervention type, diagnosis and setting) and implementing vote counting to identify significant results using ticks and crosses and highlighting strength and direction of relationships using different colours within tables (iii) Explored relationships within and between studies using conceptual mapping/idea webbing to develop a visual representation of the results of included studies (iv) Assessing the robustness of the synthesis using MMAT quality assessment tool, by assessing the quality of each paper individually, observing which MMAT criteria was met, and utilizing this information when synthesising the studies.

### 2.6. Theory of change

To develop a preliminary theory of change, outcomes were extracted according to CHIME and mental health symptoms. Studies were grouped by intervention type. In the studies included in the review, intervention type mapped onto population and resources used. Studies were extracted for reported potential mechanisms for change by two researchers.

### 2.7. Reflexivity

We are a team of multi-disciplinary team of researchers from various research and clinical backgrounds. Dave Chawner is a stand-up comedian with lived experience of an ED, who has developed and facilitated a ‘Comedy for Coping’ course for those with EDs. The idea for producing a systematic review on comedy interventions for those with mental health issues was inspired by Dave’s work, as we saw a necessity to synthesise the current evidence on this subject. Half of the research team are involved in a research project evaluating the ‘Comedy for Coping’ course. Dieter Declercq is a humanities researcher and expert on comedy, with knowledge of interpretive frameworks, which has helped guide our understanding of comedy and the socio-cultural dimensions of health. Helen Brooks, Cat Papastavrou Brooks and Una Foye are mental health researchers and brought a health-sciences focus on intervention evaluation, viewing comedy as a health intervention, and framing it within theories of change. Eshika Kafle has a background working clinically with those with mental health issues, with knowledge of traditional mental health therapies and how interventions are implemented within an NHS context.

## 3. Results

### 3.1. Description of studies

Study characteristics are presented in Table 3. Overall, 17 studies were included in the systematic review, of which 13 were published studies (Gelkopf et al., 1993, 1994, 2006; Walter et al., 2007; Hirsch et al., 2010; Falkenberg et al., 2011; Konradt et al., 2013; Cai et al., 2014; Rudnick et al., 2014; Barker and Winship, 2016; Tagalidou et al., 2018, 2019; Malhotra et al., 2020) and four were unpublished, grey literature (Biggs and Stevenson, 2011, Unpublished manuscript^2^; Palmer, 2017, Unpublished manuscript^3^; Farrants, 2019, Unpublished manuscript^4^; Belcher, 2022, Unpublished manuscript^5^). 15 studies were unique studies and two studies utilised the same intervention and participant group, but utilised different outcome measures (Gelkopf et al., 1993, 1994). One study used qualitative methodology (Belcher, 2022, Unpublished manuscript, see footnote 6), one study was an RCT (Cai et al., 2014), nine studies used a quantitative non-RCT design (Gelkopf et al., 1993, 1994, 2006; Walter et al., 2007; Hirsch et al., 2010; Falkenberg et al., 2011; Konradt et al., 2013; Barker and Winship, 2016; Malhotra et al., 2020) and six studies used mixed methods (Biggs and Stevenson, 2011, Unpublished manuscript, see footnote 3; Rudnick et al., 2014; Palmer, 2017, Unpublished manuscript, see footnote 4; Tagalidou et al., 2018, 2019; Farrants, 2019, Unpublished manuscript, see footnote 5). Of the studies which included a qualitative component, one used thematic analysis (Rudnick et al., 2014). It was unclear how other studies analysed qualitative data (Biggs and Stevenson, 2011, Unpublished manuscript, see footnote 3; Palmer, 2017, Unpublished manuscript, see footnote 4; Tagalidou et al., 2018, 2019; Farrants, 2019, Unpublished manuscript, see footnote 5).

**Table 3:** Description of studies.

Author	Type of literature	Study design	Demographics	Primary diagnosis	Delivery setting	Intervention (facilitator)	Sample size	Retention	Funding
Barker and Winship (2016)	Published	Exploratory Pilot Study	Age: 39–58 100% White British 60% Female	Substance Misuse	Substance misuse services	“Laughing Matters” comedy workshop (professional comedian)	10	40%	Not reported
Belcher (2022)	Un-published	Qualitative evaluation	–	Factors affecting mental health	GP Surgery	Creating a comedy set (professional comedian)	8	100%	Not reported
Biggs and Stevenson (2011)	Un-published	Mixed-methods (Surveys, focus groups, 1 interview)	Age: M = 42.43 (11.78) 60% Female	Mental health problem	Not reported	“Universal comedy” (professional comedians)	20	–	Funded (source not reported)
Cai et al. (2014)	Published	RCT	46% Female	Schizophrenia	Inpatient	7 Humor Habits, McGhee (untrained facilitator)	30	100%	No funding
Falkenberg et al. (2011)	Published	Pilot Study	–	Depression	–	7 Humor Habits, McGhee (untrained facilitator)	6	100%	Not reported
Gelkopf et al. (1993)	Published	‘Experiment’ (with control group)	Age: M = 43.76 (13.55)	Schizophrenia	Inpatient	Watching comedy films (ward staff)	34	65%	Not reported
Gelkopf et al. (1994)	Published	Before/After	Age: M = 45.44 (14.73) 18% Female	Schizophrenia	Inpatient	Watching comedy films (ward staff)	34	–	Not reported
Gelkopf et al. (2006)	Published	Non-randomised controlled study	Age M = 44.23 (7.98) 62% Female	Schizophrenia	Inpatient	Watching comedy films (ward staff)	29	–	Not reported
Farrants (2019)	Un-published	Project report	–	Under an NHS mental health trust	NHS Recovery College	Two courses: Improvisation and stand-up (comedian, physical theatre practitioner, comedy school director)	70	–	Not reported
Hirsch et al. (2010)	Published	Experimental group and control group	Age: 61–80, M = 73.79 (6.31) 67% Female	Depression	Outpatient Clinic	Humor Group (head physician and therapist)	90	58%	Not reported
Konradt et al. (2013)	Published	Experimental group and control group	Age: M = 71.94 (5.67) 63% Female	Depression	Inpatient	7 Humor Habits, McGhee (untrained facilitator)	99	–	No external funding
Palmer (2017)	Un-published	Learning report	Age: M = 50.15 (6.10) 41% Female White or White British: 43%	Not reported	Not reported	Introduction to improvisation course (professional comedian)	53	47%	Not reported
Rudnick et al. (2014)	Published	Mixed methods Randomized Controlled Pilot Effectiveness Study	–	Diagnosis of Mental Illness	Online via skype	Stand-up comedy training (professional comedy/counsellor/lived exp. of mental illness)	36	61%	CARE-MH (CIHR and AstraZeneca)
Tagalidou et al. (2018)	Published	Single-arm pilot study	Age: 51.9 (9.67) 74.3% female	Subclinical Depression	Outpatient Clinic	7 Humor Habits, McGhee (untrained facilitator) with adaptations for modern media	35	80%	University of Salzburg (publication costs)
Tagalidou et al. (2019)	Published	RCT	Age: 24–76, M = 50.86 (13.68) 73% Female	Depression: 59% Anxiety Disorder: 19% Adjustment disorder: 22%	Outpatient Clinic	7 Humor Habits, McGhee (untrained facilitator) with adaptations for modern media	37	81%	No funding
Malhotra et al. (2020)	Published	Cross sectional, exploratory, interventional	–	–	Inpatient	Comedy movie watching	–	–	Not reported
Walter et al. (2007)	Published	Pilot Study	Age: 62–89, M = 78 65% Female	Depression and/or Alzheimer’s Disease	Inpatient	Humor therapy group (unidentified moderator)	40	100%	Not reported

Most studies included participants with a diagnosed mental health disorder, including substance misuse (Barker and Winship, 2016), Schizophrenia (Gelkopf et al., 1993, 1994, 2006; Cai et al., 2014), depression (Walter et al., 2007; Hirsch et al., 2010; Falkenberg et al., 2011; Konradt et al., 2013; Tagalidou et al., 2019), anxiety and adjustment disorder (Tagalidou et al., 2019). One study included those with subclinical depression (Tagalidou et al., 2018). Six studies (including all four of the grey literature studies) did not specify the mental health diagnosis of participants (Biggs and Stevenson, 2011, Unpublished manuscript, see footnote 3; Rudnick et al., 2014; Palmer, 2017, Unpublished manuscript, see footnote 4; Farrants, 2019, Unpublished manuscript, see footnote 5; Malhotra et al., 2020; Belcher, 2022, Unpublished manuscript, see footnote 6).

Comedy interventions were delivered in a range of settings, seven studies were conducted in inpatient settings (Gelkopf et al., 1993, 1994, 2006; Walter et al., 2007; Konradt et al., 2013; Cai et al., 2014; Malhotra et al., 2020), including all the studies on participants diagnosed with schizophrenia. Three studies were conducted in outpatient clinics (Hirsch et al., 2010; Tagalidou et al., 2018, 2019) one in a substance misuse service (Barker and Winship, 2016), one in a GP surgery (Belcher, 2022, Unpublished manuscript, see footnote 6) and one in an NHS recovery college (Farrants, 2019, Unpublished manuscript, see footnote 5). One study delivered the comedy intervention online (Rudnick et al., 2014) and two studies did not report the setting in which the intervention was delivered (Biggs and Stevenson, 2011, Unpublished manuscript, see footnote 3; Palmer, 2017, Unpublished manuscript, see footnote 4).

Studies utilized a range of comedy interventions. Five studies evaluated the ‘7 Humor Habits’ programme, which teaches humour skills to cope with life stressors, followed by development of skills outside of sessions in “home play” (McGhee, 1996) delivered by an untrained facilitator (Falkenberg et al., 2011; Konradt et al., 2013; Cai et al., 2014; Tagalidou et al., 2018, 2019). Four studies evaluated ‘comedy movie watching’, delivered by inpatient staff (Gelkopf et al., 1993, 1994, 2006; Malhotra et al., 2020). Four studies evaluated comedy workshops delivered by professional comedians, which helped participants to create, write and perform their own comedy sets (Biggs and Stevenson, 2011, Unpublished manuscript, see footnote 3; Barker and Winship, 2016; Farrants, 2019, Unpublished manuscript, see footnote 5; Belcher, 2022, Unpublished manuscript, see footnote 6), three of which were grey literature. One study investigated the use of stand-up comedy training, delivered by a comedian/counsellor, with lived experience of mental health issues (Rudnick et al., 2014). One study utilised both improvisation and stand-up comedy courses, delivered by a comedian (Farrants, 2019, Unpublished manuscript, see footnote 5) and one study utilised an improvisation course alone (Palmer, 2017, Unpublished manuscript, see footnote 4). Two studies investigated the use of a ‘humor therapy group’ delivered by a head physician/therapist and unidentified moderator (Walter et al., 2007; Hirsch et al., 2010). Only two studies had a retention rate below 50% (Barker and Winship, 2016; Palmer, 2017, Unpublished manuscript, see footnote 4). In four studies retention rates were unclear/unreported (Gelkopf et al., 1994, 2006; Biggs and Stevenson, 2011, Unpublished manuscript, see footnote 3; Farrants, 2019, Unpublished manuscript, see footnote 5).

Three studies were funded (Biggs and Stevenson, 2011, Unpublished manuscript, see footnote 3; Rudnick et al., 2014; Tagalidou et al., 2018). Three studies were not funded (Konradt et al., 2013; Cai et al., 2014; Tagalidou et al., 2019). 11 studies did not report whether they were funded or not (Gelkopf et al., 1993, 1994, 2006; Walter et al., 2007; Hirsch et al., 2010; Falkenberg et al., 2011; Cai et al., 2014; Barker and Winship, 2016; Farrants, 2019, Unpublished manuscript, see footnote 5; Malhotra et al., 2020; Belcher, 2022, Unpublished manuscript, see footnote 6). One study was facilitated by a comedian with lived experience of mental health issues (Rudnick et al., 2014). However, it was unclear whether anyone with lived experience was involved in any other aspect of the research.

### 3.2. Participant demographics

The sample size of the included studies ranged from six to 99, M = 39.44 (26.06), with a total of sample size of 631 across 16 studies. One study did not report their sample size.

11 studies reported on the gender of participants, giving a total of 58.56% female participants across all 11 studies (279 out of 477 participants.) Only two studies reported on participants’ race; one had 100% white participants (ten participants in total) (Barker and Winship, 2016), and the other (Palmer, 2017, Unpublished manuscript, see footnote 4) was 43% White or White British, 14% Black or Black British, 5% Asian or Asian British, 10% mixed heritage and 29% not stated. 11 studies reported some information on the age of participants, however one study just stated that the range was 39–58. Of the remaining studies, the mean age (or the approximate mean age calculated from frequency tables) fell between 42.43 (Biggs and Stevenson, 2011, Unpublished manuscript, see footnote 3) and 78 (Walter et al., 2007) (for study explicitly looking at a geriatric population.)

### 3.3. Quality appraisals

Quality appraisals were assessed using the Mixed Methods Appraisal Tool (MMAT) (Hong et al., 2018). An overview of which MMAT criteria were met by each study is shown in Table 4. It is recommended to use MMAT to provide a detailed overview of the quality of studies rather than generate an overall score (Hong et al., 2018). Two studies met all MMAT criteria (7, 8) and one study did not meet any MMAT criteria (14).

**Table 4:** Quality appraisals.

MMAT Quality Appraisals Criteria																						
	S1	S2	1.1	1.2	1.3	1.4	1.5	2.1	2.2	2.3	2.4	2.5	3.1	3.2	3.3	3.4	3.5	5.1	5.2	5.3	5.4	5.5
Barker and Winship (2016)	Y	Y											N	Y	–	N	N					
Belcher (2022), Unpublished manuscript, see footnote 6	Y	N	–	N	N	N	N															
Biggs and Stevenson (2011), Unpublished manuscript, see footnote 3	Y	Y																N	Y	N	Y	N
Cai et al. (2014)	Y	Y						N	Y	Y	–	–										
Falkenberg et al. (2011)	Y	Y											Y	Y	Y	N	Y					
Farrants (2019), Unpublished manuscript, see footnote 5	Y	Y																N	N	N	Y	N
Gelkopf et al. (1994)	Y	Y											Y	Y	Y	N	Y					
Gelkopf et al. (1993)	Y	Y											Y	Y	–	N	Y					
Gelkopf et al. (2006)	Y	Y											Y	Y	–	N	Y					
Hirsch et al. (2010)	Y	Y											Y	Y	Y	Y	Y					
Konradt et al. (2013)	Y	Y											Y	Y	Y	Y	Y					
Malhotra et al. (2020)	Y	Y											N	N	-	N	Y					
Palmer (2017), Unpublished manuscript, see footnote 4	Y	Y																N	N	N	Y	N
Rudnick et al. (2014)	Y	Y																Y	N	N	N	N
Tagalidou et al. (2018)	Y	Y																Y	N	N	Y	N
Tagalidou et al. (2019)	Y	Y																Y	Y	Y	Y	N
Walter et al. (2007)	Y	Y											Y	Y	Y	N	Y					

N, no; Y, yes; –, unsure.

It is notable that the majority of studies included in this review were quantitative non-RCT studies. For several of these studies, we were unable to assess whether there was complete outcome data due to authors not reporting the number of participants who completed outcome measures (Gelkopf et al., 1993, 1994; Barker and Winship, 2016; Malhotra et al., 2020). One study included in the review was an RCT (Cai et al., 2014), however, this was judged to be of low quality, with the process by which randomisation occurred not adequately described. One study included in the review used qualitative methods (Belcher, 2022, Unpublished manuscript, see footnote 6) and was deemed to be very low quality, with collected data not adequately addressing research questions and findings not adequately derived or substantiated by the data. Several studies used mixed methods, and included a qualitative component. All unpublished literature that included a qualitative component was deemed to be of low quality. It was often unclear how qualitative findings were analysed and derived from the data and qualitative and quantitative findings were often not adequately integrated and interpreted.

### 3.4. Study outcomes

**Figure 2:**
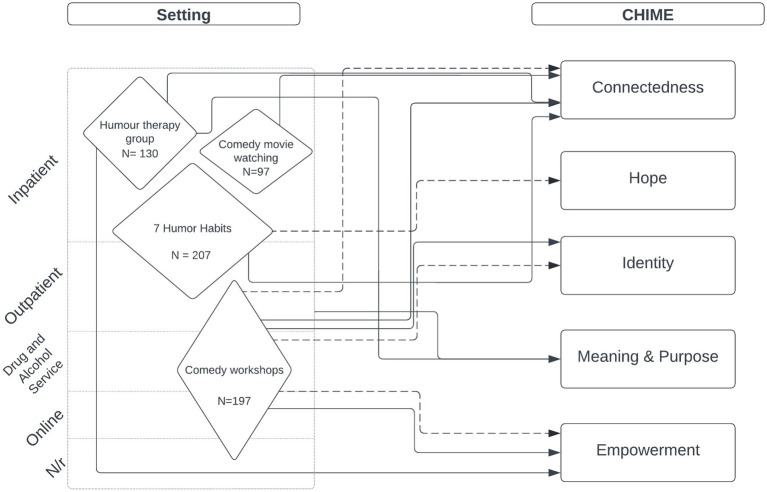
Study outcomes. Solid line denotes statistically significant difference in outcome. Dotted line shows theorised impact on outcome. Size of diamond is proportional to combined sample size in included studies.

Study outcomes were synthesised according to the CHIME framework (see Table 1), and Mental Health Outcomes. Study outcomes are outlined in Table 5 and Figure 2.

**Table 5:** Study outcomes.

	Study design	Connectedness	Hope and optimism	Identity	Meaning and purpose	Empowerment	Mental health	Acceptability
Barker and Winship (2016)	Exploratory Pilot Study	+ strength of relationship - number of relationships	+	T	T	+	+	
Belcher (2022), Unpublished manuscript, see footnote 6	Qualitative evaluation	T			T	T		
Biggs and Stevenson (2011), Unpublished manuscript, see footnote 3	Mixed-methods (Surveys, focus groups, 1 interview)	Q		Q	Q	Q	+	100% satisfaction
Cai et al. (2014)	RCT	T	T				+	
Falkenberg et al. (2011)	Pilot Study						+Short term mood NS long term mood	Patients engaged with sessions
Farrants (2019), Unpublished manuscript, see footnote 5	Project report					Q	+	Requested: longer sessions, more sensitive facilitators, more handouts
Gelkopf et al. (1993)	‘Experiment’ (with control group)	NS support from family and friends + support from staff					NS	
Gelkopf et al. (1994)	Before/After	+ support from caretakers NS support from patients						
Gelkopf et al. (2006)	Non-randomised controlled study	T				NS	+	
Hirsch et al. (2010)	Experimental group and control group	Q					+	
Konradt et al. (2013)	Experimental group and control group					+	+	
Palmer (2017), Unpublished manuscript, see footnote 4	Learning report	Q	Q	Q		Q	+	
Rudnick et al. (2014)	Mixed methods Randomized Controlled Pilot Effectiveness Study	+	+			+	NS	Logistical challenges related to participation
Tagalidou et al. (2018)	Single-arm pilot study						+	Very high satisfaction Requested: more practical games and exercises and more time
Tagalidou et al. (2019)	RCT		NS				NS	Good overall satisfaction with training
Malhotra et al. (2020)	Cross sectional, exploratory, interventional	+					Q	Reported screening of comedy movies should be a regular activity
Walter et al. (2007)	Pilot Study					+	+	

+ = Effect in the hypothesized direction, − = effect in the non-hypothesized direction, NS = non-significant, Q = qualitative, T = theorised but not present in the data.

#### 3.4.1. Connectedness

Six published studies reported improvements in connectedness due to participating in the comedy intervention (Gelkopf et al., 1994, 2006; Cai et al., 2014; Rudnick et al., 2014; Barker and Winship, 2016; Malhotra et al., 2020). Of these studies, one was an RCT (Cai et al., 2014), four were non-RCT studies (Gelkopf et al., 1994, 2006; Barker and Winship, 2016; Malhotra et al., 2020) and one was a mixed methods RCT study (Rudnick et al., 2014). This included statistically significant improvements in the strength and mutuality of relationships, as measured by eco mapping (Barker and Winship, 2016). However, a decrease in the number of relationships participants had was found (Barker and Winship, 2016). Additionally, Barker and Winship (2016) improvements in social competence (Gelkopf et al., 2006), increased perceived support from staff though not from other patients (Gelkopf et al., 1994) improvements in bonding with both staff and other patients (Malhotra et al., 2020) and benefits in social interaction and befriending, in one low quality RCT, (Rudnick et al., 2014) were found. One RCT study (Cai et al., 2014) theorised that humour created opportunities for social exchange, but did not measure this. Processes of randomisation were also inadequately reported upon and the RCT component of the study was deemed of low quality. It is worth noting that although the study which reported no change in perceived support from other patients (Gelkopf et al., 1994) attributed this to the “impoverished” social relationships between patients, the intervention (which involved watching comedy films together) could have worked by improving staff attitudes towards patients, not by enabling patients to connect better with staff. Of these studies, it is notable that few met the majority of MMAT criteria (Gelkopf et al., 1994, 2006) and were considered to be of sufficient methodological quality and only one study (Gelkopf et al., 2006) had complete outcome data.

Improvements in connectedness were also found in three unpublished reports and studies (Biggs and Stevenson, 2011, Unpublished manuscript, see footnote 3; Palmer, 2017, Unpublished manuscript, see footnote 4; Belcher, 2022, Unpublished manuscript, see footnote 6). Two of these used mixed methods (Biggs and Stevenson, 2011, Unpublished manuscript, see footnote 3; Palmer, 2017, Unpublished manuscript, see footnote 4) and one study used qualitative methods (Belcher, 2022, Unpublished manuscript, see footnote 6). Participants reported making friends at the comedy course, which had a positive impact on their lives and enabled them to develop the skills to meet new people, and providing them with additional support (Biggs and Stevenson, 2011, Unpublished manuscript, see footnote 3). A case study in one report (Palmer, 2017, Unpublished manuscript, see footnote 4) described the positive impact of having someone in the course who said “I know how you are feeling, I feel it too,” which led to decreased social anxiety, increased willingness to engage with others, and resulting improvements to mental health. One report (Belcher, 2022, Unpublished manuscript, see footnote 6) stated that the group provided support for participants, and also that the intervention encouraged participants to engage in the community, however the only evidence provided of this impact was that one participant reported that the group was “absolutely brilliant.” The quality of the qualitative component in all studies was found to be low.

#### 3.4.2. Hope

Four published studies reported some improvements in outcomes related to participant’s sense of hope, although none measured hope directly (Konradt et al., 2013; Rudnick et al., 2014; Barker and Winship, 2016; Tagalidou et al., 2019). One study was an RCT (Tagalidou et al., 2019), one study was a mixed methods RCT (Rudnick et al., 2014) and one study was non-RCT (Barker and Winship, 2016). Related outcomes included qualitatively reported increases in positive or “at least more positively balanced” thinking found in interviews with participants (Rudnick et al., 2014) and significant increases in dispositional optimism (Barker and Winship, 2016) and cheerfulness (Konradt et al., 2013; Tagalidou et al., 2019). Of these studies, one was deemed high quality and met all MMAT criteria (Konradt et al., 2013).

One unpublished report (Palmer, 2017, Unpublished manuscript, see footnote 4), which used mixed methods, described a case study, where a participant said that when they feel anxious or in a low mood they “just impro my way through it! There’s nothing to fear, just go for it!”

#### 3.4.3. Identity

No published studies directly measured a potential impact on participants sense of identity. However one low quality non-RCT study (Barker and Winship, 2016) theorised that decreases in social network strength found could be due to an adoption of a stronger recovery identity (in the context of substance misuse), leading participants to sever ties with existing social networks.

One mixed methods unpublished report (Biggs and Stevenson, 2011, Unpublished manuscript, see footnote 3) reported that the comedy course helped participants feel like “themselves” again, and that they had regained their sense of identity. Another mixed methods report (Palmer, 2017, Unpublished manuscript, see footnote 4) contained participant0 feedback that “Impro bought out a side of me that’s been hidden my entire life.”

#### 3.4.4. Meaning and purpose

Notably, no studies included any outcomes or qualitative analysis which was related to participants’ sense of meaning or purpose.

#### 3.4.5. Empowerment

Four studies reported outcomes related to empowerment (Gelkopf et al., 2006; Rudnick et al., 2014; Barker and Winship, 2016). Three studies were non-RCT (Gelkopf et al., 2006; Hirsch et al., 2010; Barker and Winship, 2016) and one study used mixed methods (Rudnick et al., 2014). One study (Barker and Winship, 2016) found an increase in self-esteem and self-efficacy and another (Gelkopf et al., 2006) found no change in daily life function. Notably, one high quality study found an increase in resilience in participants with moderate to severe depression (Hirsch et al., 2010). One study found significant increases in self-esteem, corroborated by qualitative findings, as participants reported an improvement in stigmatization of people with mental health issues (Rudnick et al., 2014).

A low quality qualitative grey literature study/report (Belcher, 2022, Unpublished manuscript, see footnote 6) stated that participants would feel empowered to try out their new skills and increase their resilience and ‘self-power’ though again evidence for this was not provided, and a second report found that participants reported that taking part in the course helped reduce the stigma they felt and that other people saw them “differently” after (Biggs and Stevenson, 2011, Unpublished manuscript, see footnote 3).

### 3.5. Symptoms and wellbeing

Several published studies found statistically significant improvements on psychological wellbeing and mood post-intervention (Walter et al., 2007; Hirsch et al., 2010; Konradt et al., 2013; Cai et al., 2014; Barker and Winship, 2016; Tagalidou et al., 2018). Five studies were non-RCT (Walter et al., 2007; Hirsch et al., 2010; Falkenberg et al., 2011; Konradt et al., 2013; Barker and Winship, 2016) and one study used mixed methods (Tagalidou et al., 2018). Six published studies found some improvement in mental health symptoms (Gelkopf et al., 2006; Walter et al., 2007; Falkenberg et al., 2011; Konradt et al., 2013; Cai et al., 2014; Tagalidou et al., 2018), including improvements in depression, anxiety and schizophrenia symptoms. Two published studies, one which used a mixed methods design (Rudnick et al., 2014) and one which used a non-RCT design (Gelkopf et al., 1993) found no improvement in mental health symptoms. One high quality semi-randomised study found improvement in depression symptoms in both control and humour therapy group (Konradt et al., 2013), but additional benefits in life satisfaction for the intervention group only. Another high quality, non-RCT study, found significant decreases in state seriousness and bad mood and an increase in quality of life for those with moderate to severe depression post-intervention (Hirsch et al., 2010). There were no qualitative findings related to mental health symptom reduction or wellbeing in the published studies. It is notable that one low quality RCT study found a significant reduction in short-term negative symptoms of depression/anxiety but found no long-term improvement in mood following the intervention (Cai et al., 2014).

Three unpublished reports found significant improvements on psychological wellbeing and mood post-intervention (Biggs and Stevenson, 2011, Unpublished manuscript, see footnote 3; Palmer, 2017, Unpublished manuscript, see footnote 4; Farrants, 2019, Unpublished manuscript, see footnote 5). All three reports used mixed methods. No unpublished studies reported any significant improvements in mental health symptoms. One unpublished study found improvements in mental health condition management (Biggs and Stevenson, 2011, Unpublished manuscript, see footnote 3).

### 3.6. Acceptability

Seven studies included data on acceptability (Biggs and Stevenson, 2011, Unpublished manuscript, see footnote 3; Falkenberg et al., 2011; Rudnick et al., 2014; Tagalidou et al., 2018, 2019; Farrants, 2019, Unpublished manuscript, see footnote 5; Malhotra et al., 2020). One study reported that all 19 participants were satisfied with the comedy intervention (Biggs and Stevenson, 2011, Unpublished manuscript, see footnote 3). In another study, authors reported that patients were willing to get involved and played an active part in the intervention (Falkenberg et al., 2011). Two studies reported high satisfaction with the intervention, as rated on a scale from one to five (Tagalidou et al., 2018, 2019), with one study reporting that participants would recommend the intervention to others (Tagalidou et al., 2019). In one study, patients expressed that screening of comedy movies should be a regular activity (Malhotra et al., 2020). In two studies participants mentioned improvements for the intervention (Tagalidou et al., 2018, 2019), such as longer sessions, more sensitive facilitators and more handouts (Farrants, 2019, Unpublished manuscript, see footnote 5). Participants also wanted more practical games and exercises in the intervention (Tagalidou et al., 2018).

### 3.7. Theory of change

**Figure 3:**
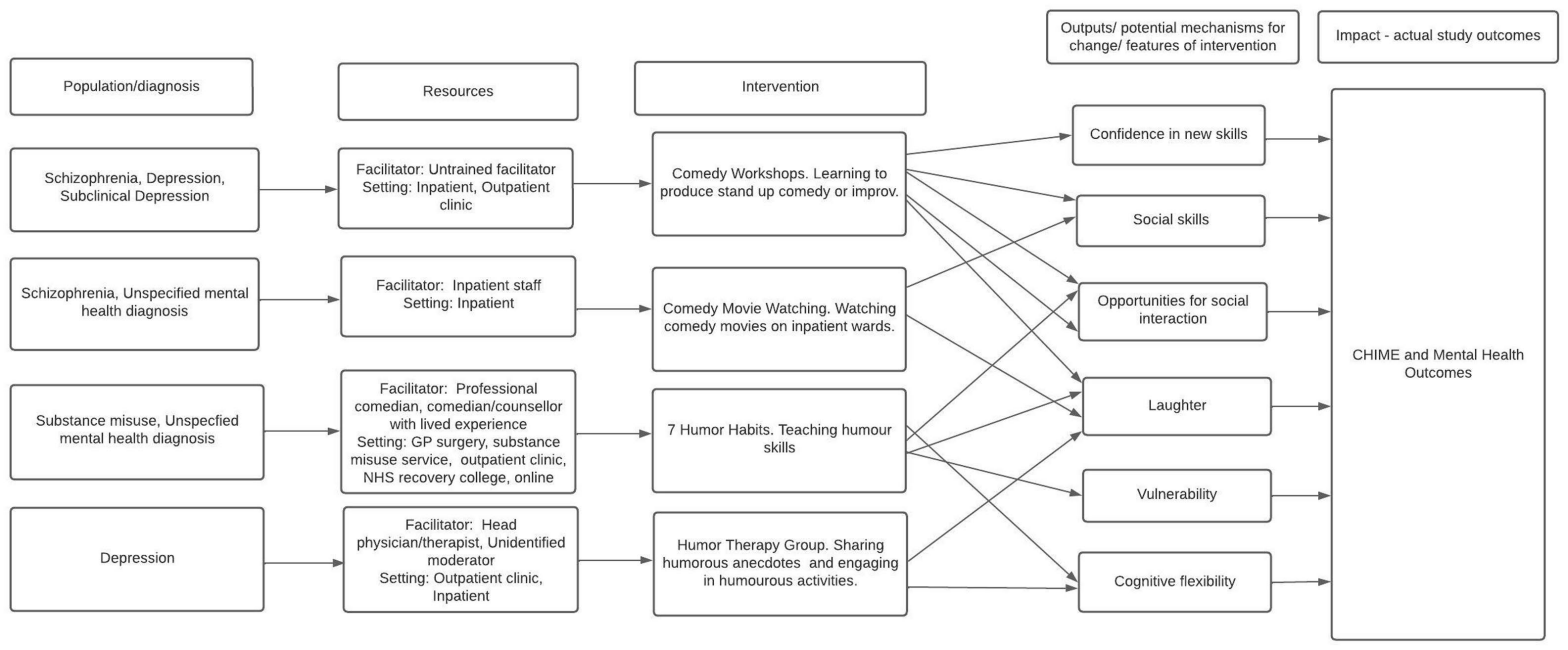
Theory of change.

There were several mechanisms proposed by studies to impact on study outcomes. Mechanisms outlined in Figure 3 are theorised to have impact on study outcomes. All lines on diagram denote theorised links.

All interventions were theorised to have impact through laughter, which was thought to reduce tension or aggression (Gelkopf et al., 1993; Walter et al., 2007; Cai et al., 2014) and increase sense of enjoyment (Rudnick et al., 2014). The ‘7 Humor Habits’ programme, which helps to increase ‘humor skills’ and use humour to cope with life stressors, may promote cognitive flexibility. For example, several studies framed humour as a ‘tool’ for coping with stressful life events, which would help participants see negative life events in a more positive light (Cai et al., 2014; Tagalidou et al., 2019). Only one study also reported that the use of humour may help participants to become more transparent about negative emotions, allowing for vulnerability (Konradt et al., 2013).

Comedy movie watching, in which comedy films were shown to those on inpatient mental health wards, was thought to have impact on mental health and CHIME outcomes due to increasing social skills and providing increased opportunities for social interaction. It was theorised that the group setting in which movies were watched gave rise to increased social exchange (Gelkopf et al., 2006) and group bonding (Gelkopf et al., 1994) in those with Schizophrenia. Notably, a higher level of support from staff was perceived to be received in one study however bonding among participants did not significantly increase as a result of the intervention (Gelkopf et al., 1993). Comedy movie watching may instead have positive impact through promoting staff-patient bonding in inpatient settings.

Comedy workshops, which taught participants to produce stand-up comedy sets or improv, were thought to have impact through increasing social skills and providing opportunities for social connection. Humour was thought to improve communication skills (Barker and Winship, 2016; Farrants, 2019, Unpublished manuscript, see footnote 5) and enable conflict situations to be resolved (Farrants, 2019, Unpublished manuscript, see footnote 5). Only comedy workshops were theorised to promote confidence in development of new skills as a mechanism for change. Comedy workshops were thought to help participants to build their self-esteem and learn skills in presentation and self-confidence (Farrants, 2019, Unpublished manuscript, see footnote 5), which may encourage employment (Biggs and Stevenson, 2011, Unpublished manuscript, see footnote 3).

Humour therapy group, in which participants tell humorous life stories and get involved in humorous activities, was thought to have impact through encouraging laughter (Walter et al., 2007) and using mixed methods. No unpublished studies reported any significant improvements in mental health symptoms. One unpublished study found improvements in mental health condition management (Biggs and Stevenson, 2011, Unpublished manuscript, see footnote 3).

## 4. Discussion

This review found that a wide range of comedy interventions have been used in a variety of settings, with varying mental health populations. Studies were found to have positive impact on several mechanisms of the CHIME framework for recovery, including connectedness, hope, identity and empowerment. No evidence was found for studies supporting the meaning and purpose component of CHIME. Further, evidence was found for the positive impact of comedy interventions on mental health symptoms (Gelkopf et al., 2006; Walter et al., 2007; Falkenberg et al., 2011; Cai et al., 2014; Tagalidou et al., 2018) and wellbeing (Walter et al., 2007; Hirsch et al., 2010; Falkenberg et al., 2011; Barker and Winship, 2016; Tagalidou et al., 2018). Comedy interventions were found to be acceptable in a number of studies (Biggs and Stevenson, 2011, Unpublished manuscript, see footnote 3; Falkenberg et al., 2011; Rudnick et al., 2014; Tagalidou et al., 2018, 2019; Farrants, 2019, Unpublished manuscript, see footnote 5; Malhotra et al., 2020). Potential theorised mechanisms for change included confidence in new skills, promotion of social skills, opportunities for social interaction, laughter, vulnerability and cognitive flexibility.

Overall, the studies provided limited detail about the content of the comedy interventions or exactly how participants engaged with the multi-faceted dimensions of comedy in the interventions. Instead, the studies focused on how results post-intervention compared to measures identified pre-intervention, generally omitting detail of what exactly happened in between. As a result, the comedy interventions remain largely a “black box.” In line with MRC guidance on evaluation and development of complex interventions, research should shed light upon which specific components of interventions have an impact on mental health (for specific people, in specific circumstances), rather than focus solely on whether interventions are effective (Skivington et al., 2021). It is recommended that interventions should be reported using the Template for Intervention Description and Replication (TIDieR) checklist to ensure interventions can be replicated and reliably implemented (Hoffmann et al., 2014). It is evident that although there is preliminary evidence for the effectiveness of comedy interventions, the current picture created by research in this area, does not outline mechanisms of impact clearly.

To unpack the black box of comedy, there is a need for more studies that incorporate robust qualitative methods in their research design. The studies reported results of various interventions, including watching comedy films (Gelkopf et al., 1993, 1994, 2006; Malhotra et al., 2020), doing stand-up comedy and improv (Biggs and Stevenson, 2011, Unpublished manuscript, see footnote 3; Barker and Winship, 2016; Farrants, 2019, Unpublished manuscript, see footnote 5; Belcher, 2022, Unpublished manuscript, see footnote 6), and humour therapy groups (Falkenberg et al., 2011; Konradt et al., 2013; Cai et al., 2014; Tagalidou et al., 2018, 2019). These various interventions are different in content and may elicit very different kinds of comedy-related engagement (e.g., enjoying vs. doing comedy) (Martin and Kuiper, 2016).

According to Martin (2004), comedy has different dimensions that participants can engage with including a social dimension (e.g., joking together); a cognitive dimension (e.g., getting the joke); emotional dimension (e.g., feeling amused by a joke); and a physical dimension (e.g., laughing at a joke). Comedy may be framed as a tool, which has certain affordances (Declercq, 2021) that lend themselves to improving mental health. For example, in the current study, only stand-up comedy workshops were theorised to have impact on wellbeing via increasing confidence in skills. Many studies were found to have impact on connectedness, however, other aspects of CHIME, including meaning and purpose, were not found to be impacted by comedy interventions. A more robust integration of qualitative methods in future studies is required to uncover the multi-faceted components of change in various comedy interventions.

Further, studies were found to be conducted in a wide range of contexts, e.g., inpatient (Gelkopf et al., 1993, 1994, 2006; Walter et al., 2007; Konradt et al., 2013; Cai et al., 2014; Malhotra et al., 2020) and community settings (Hirsch et al., 2010; Tagalidou et al., 2018, 2019) and online (Rudnick et al., 2014), with a range of mental health populations. Many studies did not specify the mental health diagnosis of participants (Biggs and Stevenson, 2011, Unpublished manuscript, see footnote 3; Rudnick et al., 2014; Palmer, 2017, Unpublished manuscript, see footnote 4; Farrants, 2019, Unpublished manuscript, see footnote 5; Malhotra et al., 2020; Belcher, 2022, Unpublished manuscript, see footnote 6) or provide information on how interventions were implemented. There is a need to understand how contextual factors may impact outcomes of interventions (Skivington et al., 2021). Therefore, further research is required to investigate the interaction between context and comedy interventions and its impact on mental health outcomes.

MRC guidance on development of complex interventions suggests that key stakeholders within interventions are involved in its development (Skivington et al., 2021). This systematic review found that in one study the comedy intervention was facilitated by a comedian with lived experience of mental health issues (Rudnick et al., 2014). However, it was not outlined whether, and in what capacity, the voice of those with lived experience of mental health issues was involved in evaluation of the intervention, in the study.

There have been calls for increased co-production (involvement of those with lived experience of mental health issues) in the development of public health interventions, as this can maximise acceptability, feasibility and quality of interventions, ensure selection of appropriate outcome measures and ensure interventions meet the needs of the populations they serve (Hawkins et al., 2017). Furthermore, the grey literature included in this review highlights that there is an alternative circuit of comedy interventions led by professional comedians in charitable, commercial and sometimes clinical settings. Crucially, the findings of these interventions typically do not find their way into dissemination avenues for academic research. Concurrently, the lack of robust methodologies would prevent these studies from passing though peer-review (Moher et al., 2014).

Collaboration amongst comedians, those with lived experience of mental health issues and researchers is in line with a Transdisciplinary Action Research (TDAR) approach, in which researchers from various disciplines, and key stakeholders, come to a joint understanding about social problems and interventions used to target them (Eldredge et al., 2016). Professional comedians may have an intimate understanding of the affordances of comedy as tools, whereas psychologists and psychiatrists have expertise on mental health and methodology. By combining their expertise, future studies stand to become richer and more robust.

### 4.1. Strengths, limitations and future directions

This systematic review was conducted using a rigorous search strategy and extraction methods. All studies were screened independently by two reviewers, to prevent bias. Extraction was also conducted independently, using a pre-created framework, with 30% of studies extracted by two reviewers. All conflicts were settled via discussion between reviewers. Both published and unpublished literature was included in the review, allowing for a comprehensive understanding to be gathered of the current data available on comedy interventions delivered in a wide range of settings.

Overall, quality of the included studies was low, with only two studies considered to be of high quality. In particular, there was a lack of high quality RCTs and qualitative research investigating comedy interventions was poorly conducted. Due to the lack of qualitative research conducted on comedy interventions, it was difficult to determine potential mechanisms of change and all mechanisms of change were theorised. Additionally, only 11 out of 17 included studies reported on participant demographics. We therefore have little idea about who benefits from comedy interventions and whether these interventions are beneficial for minoritized groups. The preliminary theory of change outlined in our paper should serve as a platform for the evaluation of future comedy interventions and allow hypotheses to be developed and tested. This will allow for interrogation and refinement of the theory of change. It is important that given potential benefits of comedy interventions for those with mental health conditions, high quality research in this area is conducted in order to build an evidence base for policy makers and commissioners.

## 5. Conclusion

In conclusion, some promising evidence has been found for the efficacy of a range of comedy interventions on mental health. This evidence was mostly deemed to be of low quality. There is a need for greater research input in this area, to uncover the wide range of mechanisms which may lead to positive outcomes for mental health recovery and wellbeing. Further research should involve high quality qualitative research, to explore the impact of specific components of different comedy interventions on different mental health populations and within different mental health settings. Further, there is a need for greater transdisciplinary collaboration and co-production in this area, to aid development of a richer understanding of the benefits of comedy interventions for mental health.

## Data availability statement

Publicly available datasets were analyzed in this study. This data can be found here: Protocols and searches are available at https://www.crd.york.ac.uk/PROSPERO/display_record.php?RecordID=301750. All other data will be made available by the authors, without undue reservation.

## Author contributions

EK wrote the majority of manuscript, conducted quality assessments, narrative synthesis and produced theory of change. CPB conducted quality assessments, narrative synthesis and produced theory of change. EK, CPB, DC, UF, DD, and HB were responsible for writing manuscript, reviewing studies for eligibility and data extraction and decided the study protocol (including inclusion or exclusion criteria and search strategy). DC and HB edited the manuscript. All authors contributed to the article and approved the submitted version.

## Conflict of interest

DC is a stand-up comedian with lived experience of an eating disorder, who has developed and facilitated a stand-up comedy course for those with eating disorders, called Comedy for Coping.

The remaining authors declare that the research was conducted in the absence of any commercial or financial relationships that could be construed as a potential conflict of interest.

## Publisher’s note

All claims expressed in this article are solely those of the authors and do not necessarily represent those of their affiliated organizations, or those of the publisher, the editors and the reviewers. Any product that may be evaluated in this article, or claim that may be made by its manufacturer, is not guaranteed or endorsed by the publisher.

## Footnotes

^1^ https://www.Covidence.org

^2^Biggs, and Stevenson. (2011). Universal comedy. [Unpublished manuscript].

^3^Palmer, K. (2017). Comedy school. [Unpublished manuscript].

^4^Farrants. (2019). The wellbeing project. [Unpublished manuscript].

^5^Belcher, A. (2022). Wellspring project. [Unpublished manuscript].
