# Corrigendum: “Beyond laughter”: a systematic review to understand how interventions utilise comedy for individuals experiencing mental health problems

**DOI:** 10.3389/fpsyg.2023.1328423

**Published:** 2023-11-15

**Authors:** Eshika Kafle, Cat Papastavrou Brooks, Dave Chawner, Una Foye, Dieter Declercq, Helen Brooks

**Affiliations:** ^1^School of Arts, University of Kent, Canterbury, United Kingdom; ^2^Sussex Partnership Innovation and Research in Eating Disorders (SPIRED) Clinic, Sussex Partnership Foundation Trust, Sussex, United Kingdom; ^3^Bristol Medical School, University of Bristol, Bristol, United Kingdom; ^4^Department of Mental Health Nursing, King's College London, London, United Kingdom; ^5^Mental Health Research Group, Division of Nursing, Midwifery and Social Work, School of Health Sciences, Faculty of Biology, Medicine and Health, University of Manchester, Manchester, United Kingdom

**Keywords:** mental health, mental illness, recovery, CHIME, comedy, humour, comedy intervention, humour intervention

In the published article, there was an error. A study which was a mixed methods RCT was only noted as a mixed methods study. This was requested to be changed by the author of the original study.

A correction has been made to Section 3. Results, “*3.1 Description of studies”*, paragraph 1. The incorrect sentence previously stated:

“One study used qualitative methodology (Belcher, 2022, Unpublished manuscript, see footnote 6), one study was an RCT (Cai et al., 2014), nine studies used a quantitative non-RCT design (Gelkopf et al., 1993, 1994, 2006; Walter et al., 2007; Hirsch et al., 2010; Falkenberg et al., 2011; Konradt et al., 2013; Barker and Winship, 2016; Malhotra et al., 2020) and six studies used mixed methods (Biggs and Stevenson, 2011, Unpublished manuscript, see footnote 3; Rudnick et al., 2014; Palmer, 2017, Unpublished manuscript, see footnote 4; Tagalidou et al., 2018, 2019; Farrants, 2019, Unpublished manuscript, see footnote 5).”

The corrected paragraph appears below.

Study characteristics are presented in Table 3. Overall, 17 studies were included in the systematic review, of which 13 were published studies (Gelkopf et al., 1993, 1994, 2006; Walter et al., 2007; Hirsch et al., 2010; Falkenberg et al., 2011; Konradt et al., 2013; Cai et al., 2014; Rudnick et al., 2014; Barker and Winship, 2016; Tagalidou et al., 2018, 2019; Malhotra et al., 2020) and four were unpublished, grey literature (Biggs and Stevenson, 2011, Unpublished manuscript^2^; Palmer, 2017, Unpublished manuscript^3^; Farrants, 2019, Unpublished manuscript^4^; Belcher, 2022, Unpublished manuscript^5^). 15 studies were unique studies and two studies utilised the same intervention and participant group, but utilised different outcome measures (Gelkopf et al., 1993, 1994). One study used qualitative methodology (Belcher, 2022, Unpublished manuscript, see footnote 5), one study was an RCT (Cai et al., 2014), nine studies used a quantitative non-RCT design (Gelkopf et al., 1993, 1994, 2006; Walter et al., 2007; Hirsch et al., 2010; Falkenberg et al., 2011; Konradt et al., 2013; Barker and Winship, 2016; Malhotra et al., 2020) and six studies used mixed methods (Biggs and Stevenson, 2011, Unpublished manuscript, see footnote 2; Rudnick et al., 2014; Palmer, 2017, Unpublished manuscript, see footnote 3; Tagalidou et al., 2018, 2019; Farrants, 2019, Unpublished manuscript, see footnote 4). One of these mixed methods studies was a mixed methods RCT (Rudnick et al., 2014). Of the studies which included a qualitative component, one used thematic analysis (Rudnick et al., 2014). It was unclear how other studies analysed qualitative data (Biggs and Stevenson, 2011, Unpublished manuscript, see footnote 2; Palmer, 2017, Unpublished manuscript, see footnote 3; Tagalidou et al., 2018, 2019; Farrants, 2019, Unpublished manuscript, see footnote 4).

The authors apologize for this error and state that this does not change the scientific conclusions of the article in any way. The original article has been updated.
