# Supplementary material for: “Beyond laughter”: a systematic review to understand how interventions utilise comedy for individuals experiencing mental health problems
Source: Front Psychol. 2023 Aug 7;14:1161703. doi: 10.3389/fpsyg.2023.1161703 (PMC10442070; doi:10.3389/fpsyg.2023.1161703)
Supplement: Supplementary file 1 [file Data_Sheet_1.pdf]

Search Strategy

((Humo?r* or Comed* or Jok*) and (Mental or Wellbeing or mood or psych* or Well-being or Coping or Depressi* or Anxiet* or Emotional dis or Schizo* or Bipolar or Eating adj Disorder* or Trauma or Emotional adj difficult* or Anorexi* or Bulimi* or Personality adj Disorder* or PTSD or Phobia* or Alcohol* or Addict* or (substance *use) or (substance dependen*))
